# Supplementary material for: The evolution and mutational robustness of chromatin accessibility in Drosophila
Source: Genome Biol. 2023 Oct 16;24:232. doi: 10.1186/s13059-023-03079-5 (PMC10578003; doi:10.1186/s13059-023-03079-5)
Supplement: Supplementary file 3 — Additional file 3. Motifs predictive of chromatin accessibility. [file 13059_2023_3079_MOESM3_ESM.zip › tfmodisco/metacluster_list.rtf]

motif_category = {'inc_head': ['metacluster_1', 'metacluster_4'],                   'dec_head': ['metacluster_0', ],                   'inc_testis': ['metacluster_1', 'metacluster_3'],                  'dec_testis': ['metacluster_0', 'metacluster_2']}metacluster_0: decrease head, decrease testismetacluster_1: increase head, increase testismetacluster_2: no change head, decrease testismetacluster_3: no change head, increase testismetacluster_4: increase head, no change testis
